# Rapid, Culture-Free Detection of *Staphylococcus aureus* Bacteremia

**DOI:** 10.1371/journal.pone.0157234

**Published:** 2016-06-15

**Authors:** Elliot L. Burghardt, Katie S. Flenker, Karen C. Clark, Jeff Miguel, Dilek Ince, Patricia Winokur, Bradley Ford, James O. McNamara

**Affiliations:** 1 Department of Internal Medicine, Roy J. and Lucille A. Carver College of Medicine, University of Iowa, Iowa City, Iowa, United States of America; 2 Department of Pathology, Roy J. and Lucille A. Carver College of Medicine, University of Iowa, Iowa City, Iowa, United States of America; Universitätsklinikum Hamburg-Eppendorf, GERMANY

## Abstract

*S*. *aureus* bacteremia (SAB) is a common condition with high rates of morbidity and mortality. Current methods used to diagnose SAB take at least a day, and often longer. Patients with suspected bacteremia must therefore be empirically treated, often unnecessarily, while assay results are pending. In this proof-of-concept study, we describe an inexpensive assay that detects SAB via the detection of micrococcal nuclease (an enzyme secreted by *S*. *aureus*) in patient plasma samples in less than three hours. In total, 17 patient plasma samples from culture-confirmed *S*. *aureus* bacteremic individuals were tested. 16 of these yielded greater nuclease assay signals than samples from uninfected controls or individuals with non-*S*. *aureus* bacteremia. These results suggest that a nuclease-detecting assay may enable the rapid and inexpensive diagnosis of SAB, which is expected to substantially reduce the mortality and morbidity that result from this condition.

## Introduction

*S*. *aureus* is the leading cause of nosocomial bacteremia [1, 2] and the second leading cause of community acquired bacteremia [1]. Despite the use of appropriate antibiotics, the mortality rate for SAB remains high at approximately 20% [3]. The virulence of this pathogen is also reflected by its propensity to metastasize to various tissues, often resulting in multifocal infections that are difficult to treat [4, 5]. This potential to invade tissues is one reason that SAB is treated with longer therapeutic time-courses [6] and treatment is monitored with follow-up blood cultures to confirm treatment efficacy [7]. Additionally, the rise of methicillin-resistant *S*. *aureus* (MRSA) and the inherent delay in diagnosis of bacteremia drive empiric use of vancomycin, which is inadequate or ineffective against many bacteria and often causes kidney damage and hypotensive reactions due to mast cell degranulation.

Current diagnostic assays for bacteremia rely on time-consuming blood culture-based methods that take a day or more. Treatment of patients suspected of having bacteremia is therefore based on the empiric choice of antibiotics for a day or longer, while liquid and subsequent plate cultures are performed to identify the causative pathogen. The exceedingly low bacterial load often found in the blood of bacteremic individuals (often <1 CFU/ml blood) [8] is one of the main challenges for rapid detection. Blood culture is currently used not only to determine the presence of bacteria in blood, but also to provide a sufficient number of bacteria for determining the causative bacterial species. Existing molecular assays for detecting bacteremia directly from blood (most are PCR-based) are not widely used as they have limited or uncertain diagnostic value and lack regulatory approval by the Food and Drug Administration [9, 10]. At present, this means that most “rapid” diagnostic assays for SAB are performed only on positive blood culture bottles, which take hours to several days to obtain.

Among the older approaches described for identifying *S*. *aureus* in blood cultures that produce bacterial growth is measurement of micrococcal nuclease (MN) activity [11]; MN is specifically secreted by *S*. *aureus*. MN has no disulfide bonds [12] and can re-fold into its original, active conformation upon heat denaturation [13] and has thus also been known as thermonuclease [14]. Assays that identify *S*. *aureus* in blood cultures by measuring heat-denaturation resistant nuclease activity in positive blood cultures have been described over 50 years ago [11]. While various reports have since documented the value of this approach, nuclease activity in these studies was only measured after the bacteria were cultured [15–17].

Here, we describe a culture-independent assay that detects MN activity in the blood of patients with SAB in 3 hours.

## Results

Because *S*. *aureus* secretes MN, SAB is expected to result in some level of MN in the blood. However, the low bacterial load of bacteremic patients suggests that this level will be exceedingly low [8]. We therefore sought a highly sensitive means of detecting MN in blood. We recognized that the enzymatic activity of the nuclease could provide the foundation for an ultrasensitive assay for its detection. In particular, artificial substrates, such as quenched fluorescent oligonucleotide probes, that yield signal upon digestion, have been used for the sensitive detection of various nucleases [18]. We previously described a method for noninvasive detection of *S*. *aureus* infections via MN digestion of such a probe in mice [19]. In this case, we used a chemically modified oligonucleotide probe that is responsive to MN but also widely resistant to other nucleases. Here, to maximize sensitivity for MN, we carried out kinetics assays with variants of this probe and found a simple, unmodified poly-deoxythymidine format to be the most responsive to MN digestion (not shown).

**Fig 1:**
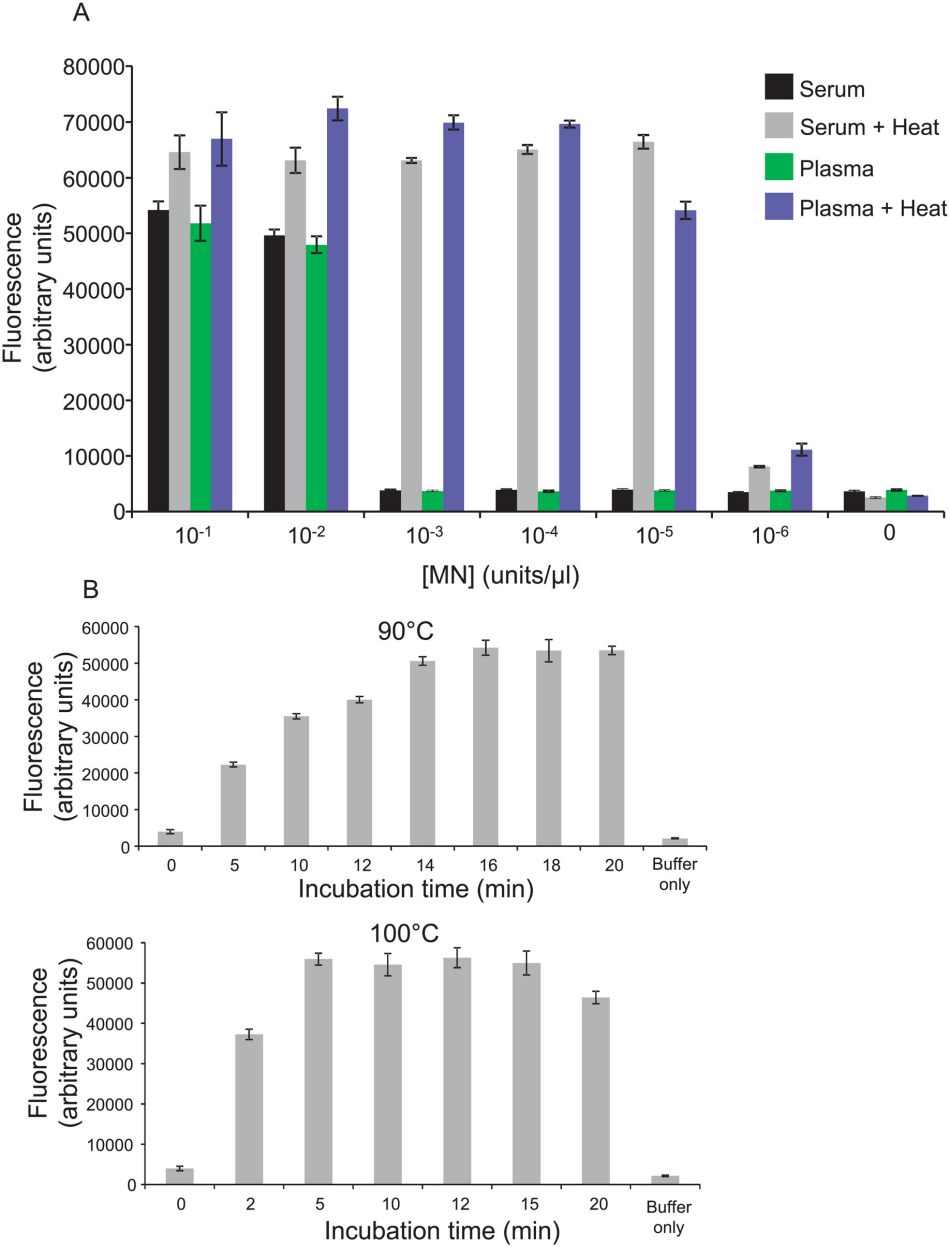
Inactivation of MN inhibitory activity with a heat treatment step. Human serum and plasma that was spiked with calcium chloride and the indicated amounts of MN was either heated to 90°C or incubated at 4°C, for 20 minutes (A). Unheated samples and supernatants of heated samples were then incubated with the 11mer PolyT FAM probe and fluorescence was measured. Note the clear differences between the heated and unheated samples with MN concentrations below 10^−2^ units/μl (panel in A). Error bars indicate standard deviations of triplicate measurements. The incubation time needed to inactivate inhibitory antibodies in plasma at 90 and 100°C was determined as follows. Human plasma, spiked with 10^−5^ units/μl MN and 10 mM calcium chloride, was heated to 90°C (upper panel in B) or 100°C (lower panel in B) for indicated amounts of time, centrifuged, and supernatants were incubated with the 11mer polyT FAM probe. Probe incubated with buffer only was included as control. Error bars indicate standard deviations of triplicate measurements.

Incubation of an 11mer polyT quenched fluorescent oligonucleotide probe (see 11mer PolyT (FAM labeled) in Table 1) with human plasma or serum that was spiked with MN yielded strong fluorescence at high concentrations of MN, but probe activation was not seen at concentrations of 10^−3^ units/μl or below (Fig 1A). MN concentrations were previously found to be between 0.1 and 1.2 units/μl in pure *S*. *aureus* cultures [20]. MN concentrations in blood of bacteremic individuals are likely much lower than this detection limit, suggesting that further improvements in assay sensitivity were needed.

**Table 1:** FAM is fluorescein amidite fluorophore; ATTO is ATTO-488 fluorophore; ZEN is IDT’s ZEN dark quencher; IAbRQ is IDT’s Iowa Black RQ dark quencher; T is the deoxythymidine nucleotide.

11mer PolyT (FAM labeled)	FAM-T-T-T-T-T-T-T-T-T-T-T-ZEN-IAbRQ
4mer PolyT (FAM labeled)	FAM-T-T-T-T-ZEN-IAbRQ
11mer PolyT (ATTO-488 labeled)	ATTO-T-T-T-T-T-T-T-T-T-T-T-ZEN-IAbRQ
4mer PolyT (ATTO-488 labeled)	ATTO-T-T-T-T-ZEN-IAbRQ

MN inhibitory antibodies are known to be present in human serum [21]. We reasoned that some means of inactivating such antibodies might enable increased MN detection sensitivity with an MN responsive probe. Because MN is known to be resistant to inactivation by heat denaturation in the presence of calcium chloride [13], we supplemented human plasma and serum with calcium chloride, added MN and heated to 90°C for 20 minutes. A dense precipitate which formed in these preparations was pelleted via centrifugation and supernatants were recovered. As shown in Fig 1A, this heat treatment yielded an increase in MN detection sensitivity of several orders of magnitude compared with no heat treatment. Time-courses of heat incubations at 90°C and 100°C revealed that this heat treatment step can be shortened to 5 minutes if the plasma is incubated at 100°C (Fig 1B).

**Fig 2:**
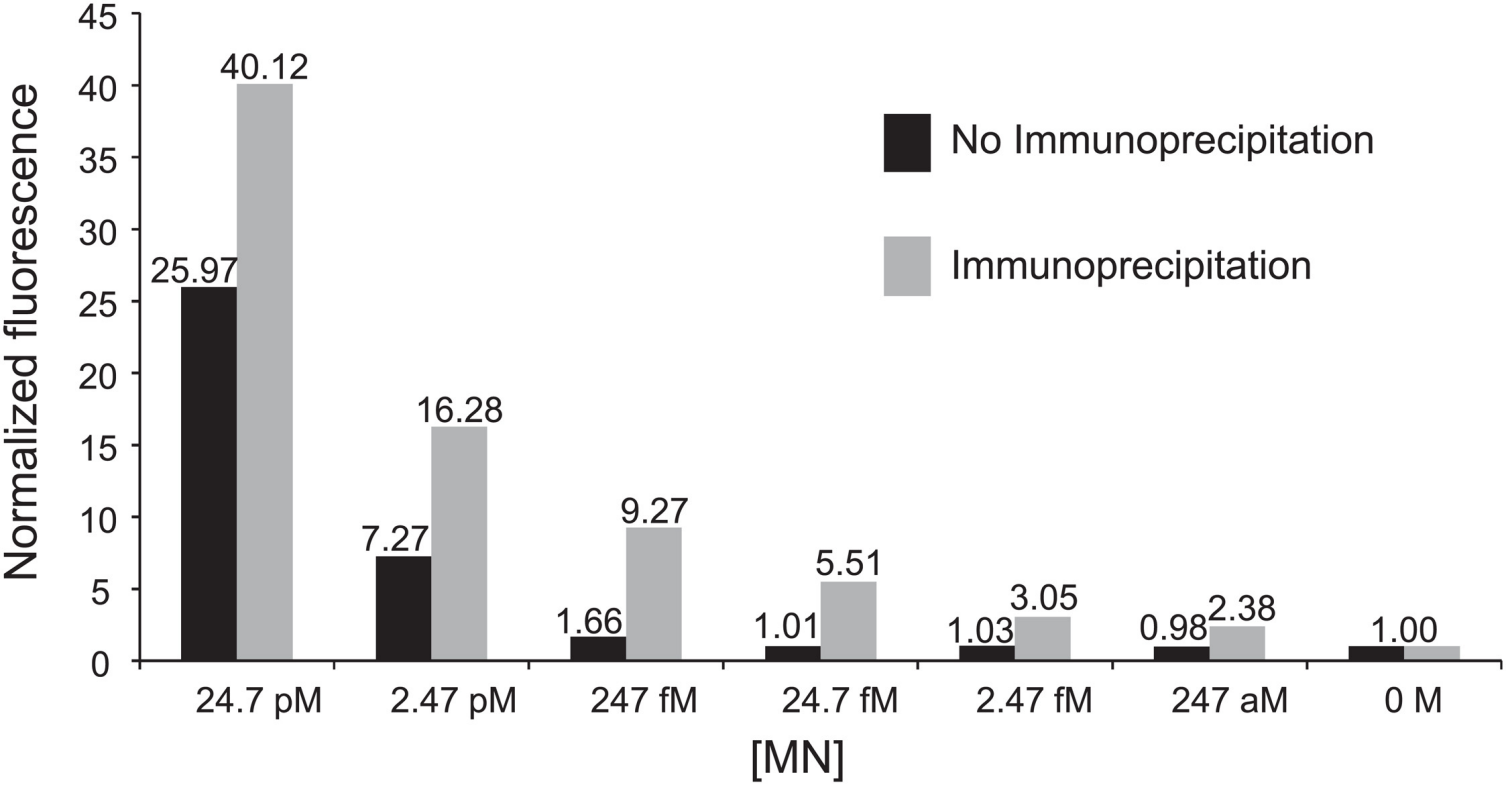
Nuclease assay detection sensitivity in human plasma. MN was immunoprecipitated from heat-treated human plasma that was spiked with MN (concentrations are indicated above), followed by incubation with a quenched fluorescent oligonucleotide probe (11mer PolyT FAM probe). Note the improvement in assay sensitivity when the immunoprecipitation step is included (gray) versus simply incubating heat-treated plasma directly with the probe (black). Fluorescence values are normalized to those of control samples in which no nuclease was included.

We considered different means of concentrating MN from heat treated plasma supernatants to further increase assay sensitivity. Immunoprecipitation was deemed an attractive choice because it is also expected to increase the specificity of the assay for MN. As suitable antibodies were not commercially available, a novel anti-MN monoclonal antibody was generated. Because the goal was not only a very sensitive, but also a rapid assay, we sought an antibody that did not need to be dissociated from MN prior to probe digestion (i.e., MN elution step could be omitted). Hybridoma clone supernatants were screened to identify hybridomas that produced antibodies that bind MN without inhibiting its enzymatic activity. A hybridoma clone that secreted an antibody with these properties was selected and used to generate a purified rat monoclonal anti-MN antibody. Protein G-coupled magnetic beads provided a means of rapid washing and concentration of the MN-coupled matrix. A simple, ~3 hour long protocol that involved incubation of MN-immunoprecipitated beads directly with probe yielded an improvement in detection sensitivity of several orders of magnitude compared with omitting the immunoprecipitation step (Fig 2). A similar assay format has previously been reported to yield ultrasensitive detection of a protease target [22].

**Fig 3:**
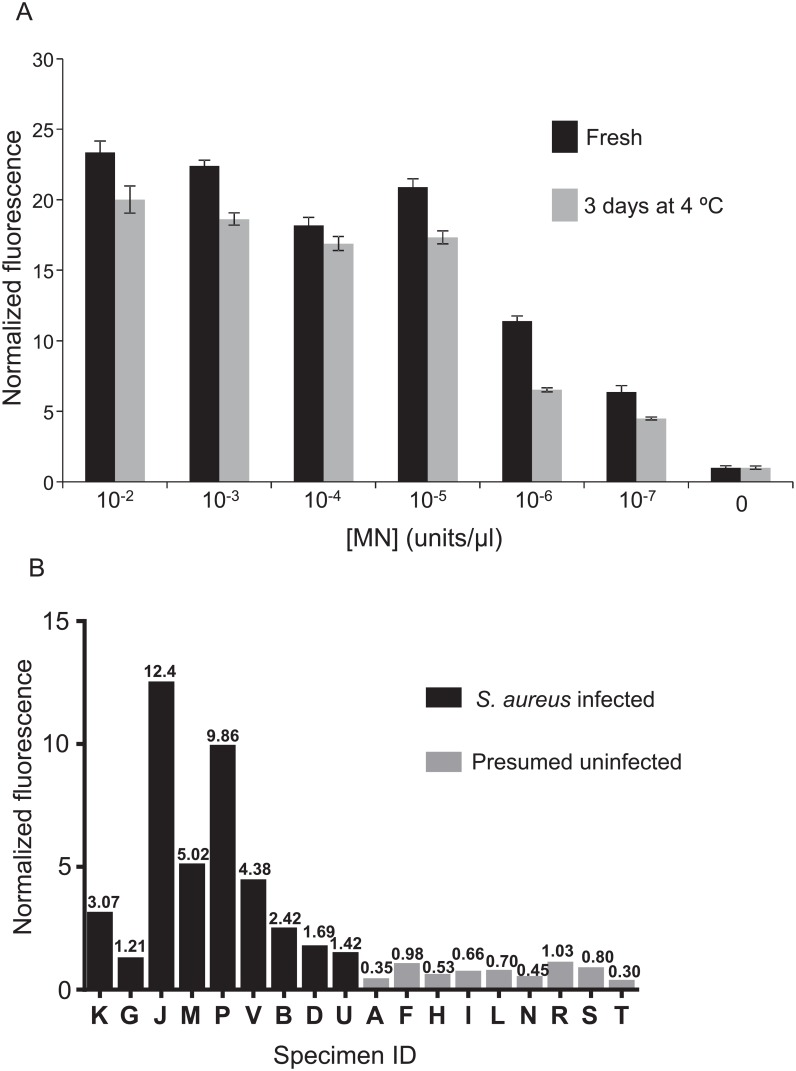
Detection of MN in SAB patient plasma. First, the stability of MN in human plasma at 4°C was determined by spiking pooled human plasma with the indicated concentrations of MN and storing at 4°C for 3 days, followed by MN activity measurement (A). Equivalent amounts of MN were also added to pooled human plasma immediately before the nuclease assay for comparison. For the assay, calcium chloride was added to all samples, which were then heated to denature inhibitory antibodies. Supernatants of heated plasma samples were incubated with the 11mer PolyT FAM probe for 1 hour and fluorescence was measured with a plate-reader. All values are normalized to those produced by control samples in which nuclease was omitted. Error bars indicate standard deviations of triplicate measurements. In part B, MN activity assays were carried out with plasma specimens from *S*. *aureus* bacteremic (*S*. *aureus* infected) and individuals showing no signs of active infections (Presumed uninfected). 660 μl of plasma was used for each and the 11mer PolyT FAM probe was used for detection. Fluorescence values were normalized to those of control samples in which buffer was substituted for plasma; note normalization values included above each bar. Data shown are compiled from several independent experiments.

We sought to determine whether this assay might be sufficiently sensitive to detect MN in plasma of individuals with SAB. To facilitate acquisition of sufficient numbers of blood samples from individuals with SAB, we planned to screen salvage plasma (stored at 4°C for several days) that was drawn on the same day as blood drawn for clinical microbiological culture-based assays. This allowed the review of clinical assay results and then selection of plasma samples that were drawn from individuals with confirmed SAB. Prior to evaluating patient samples, we measured the stability of MN diluted in human plasma and stored at 4°C for three days. As shown in Fig 3A, the activity of MN is lower in three day old mixtures compared with freshly prepared dilutions, but the loss in activity was modest.

The nuclease assay was evaluated using culture assay-confirmed *S*. *aureus* bacteremic patient plasma samples, acquired as described above (see S1 Table for sample details and culture results). Controls for this initial evaluation consisted of plasma obtained from individuals exhibiting no signs of active infection. These samples were presumed to be negative for *S*. *aureus*; blood cultures were not prepared from these individuals. Altogether, the nuclease assay signals from the nine confirmed *S*. *aureus* positive samples were all greater than those of the nine presumed negative samples (Fig 3B). With a signal threshold of 1.10, the sensitivity and specificity were 100% (versus gold-standard blood culture).

We considered the possibility that the fluorescence signal produced in the nuclease assay might provide an indication of the bacterial load in the blood. The only data from the clinical microbiological assays that is indicative of bacterial load is the time needed for the automated blood cultures to detect bacterial growth (“time-to-positivity”); shorter times generally indicate greater bacterial load. Comparing the nuclease assay signals of the bacteremic patients (Fig 3B) with the time-to-positivity values for the corresponding cultures (S1 Table) did not reveal a clear relationship between these results.

**Fig 4:**
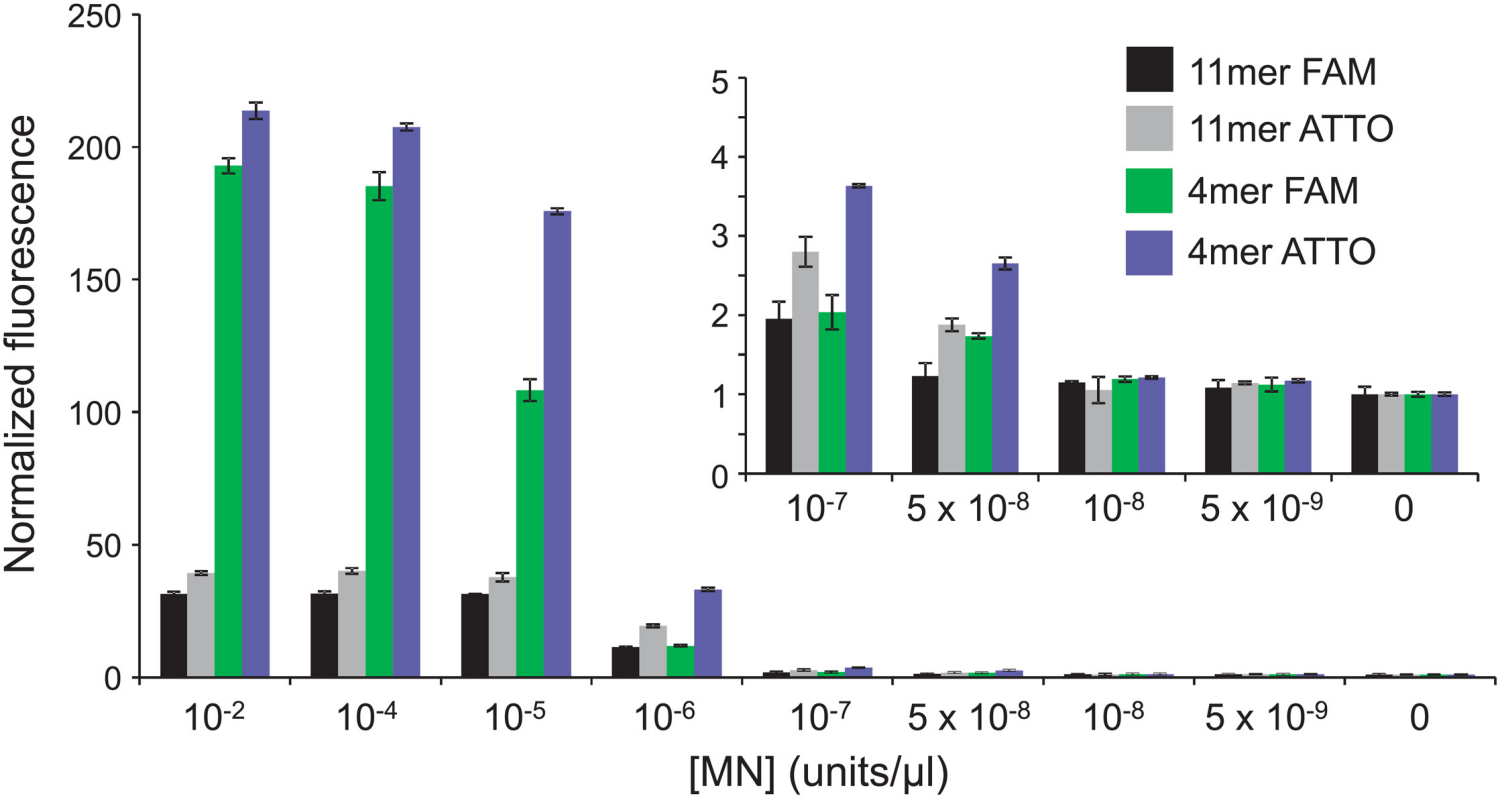
Sensitivity of MN detection with PolyT length and dye variants. Each of the indicated probes was combined with the various indicated concentrations of MN (diluted in buffer) and incubated at 37°C for 1 hour and then fluorescence was measured. Fluorescence values are normalized to those of control samples in which no nuclease was included. Fluorescence values for the lowest MN concentrations are also plotted with a different scale for better differentiation of the lower values (inset). Error bars indicate standard deviations of triplicate measurements.

To gain a more rigorous assessment of our MN-based SAB detection approach, we arranged to evaluate plasma specimens drawn from individuals who were confirmed (*via* blood culture-based assays) to have 1) no bacteremia, 2) non-*S*. *aureus* bacteremia (i.e., bacteremia with a different species), or 3) SAB (see S2 Table for sample details and culture results). For this, we used a shorter probe coupled with ATTO 488 (see 4mer PolyT (ATTO-488 labeled) in Table 1) as we found this probe to be more sensitive to MN (Fig 4); shorter probes are also less susceptible to various non-target nucleases. We also used a greater volume of plasma (2ml) as we found increased sensitivity when more plasma was used as input (not shown). To increase the number of readily accessible control samples, we revised the criteria for their selection to also include samples that were drawn from patients one day after the draw date for the available culture assay result (see far right column in S2 Table).

**Fig 5:**
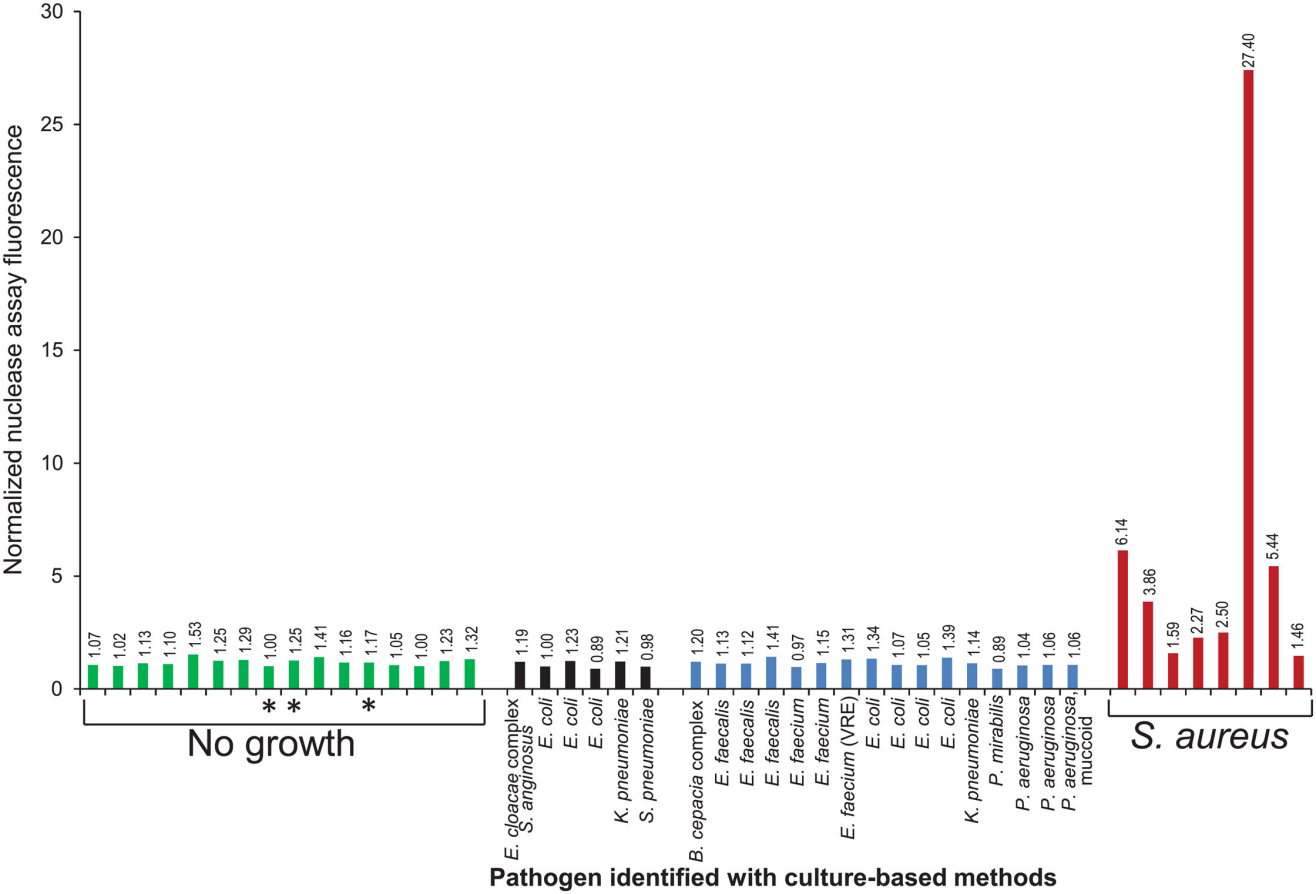
Detection of MN in plasma of patients with bacteremia. Nuclease activity assays were carried out with plasma samples from uninfected patients (No growth (green bars)), patients with positive blood culture results for non-*S*. *aureus* pathogens (black and blue bars; pathogen is indicated) or for *S*. *aureus* (red bars). Blood used for blood cultures was drawn the same day (green bars without asterisks, blue and red bars) or one day prior to (green bars with asterisks and black bars) the blood drawn for the nuclease assays. 2 ml of plasma was used for each nuclease assay and the 4mer PolyT ATTO probe was used for detection. Fluorescence values are normalized to the average fluorescence value of control samples in which buffer was substituted for plasma. Data shown are compiled from several independent experiments. Some experiments are excluded from the compilation due to failed control reactions. Culture-confirmed SAB samples for which same day culture data are unavailable are also not included due to the difficulty in interpreting these data. The researcher carrying out the nuclease assays was blinded to the results of the culture-based assays until the nuclease assay results were obtained and reviewed.

The nuclease assay signals of 7 of the 8 plasma samples drawn from individuals with confirmed SAB were greater than all of those confirmed to have no bacteremia, suggesting the assay is quite sensitive for *S*. *aureus* (Fig 5). The bacterial species making up the non-*S*. *aureus* group included several of the most common pathogens that cause bacteremia, including 7 species from 3 distinct families among the same-day culture-confirmed non-*S*. *aureus* bacteremic samples (10 species from 5 bacterial families total, when the samples from patients confirmed to be bacteremic 1 day prior to the nuclease assay sample blood draws are included). None of the plasma samples assayed from the individuals with non-*S*. *aureus* bacteremia yielded nuclease assay signals as high as those from the patients with SAB, suggesting this assay is also highly specific for *S*. *aureus*. With a signal threshold of 1.45, the sensitivity of the assay for *S*. *aureus* bacteremia is 100%; specificity is 97% (versus gold-standard blood culture). We again found no clear relationship between the nuclease assay signals and the time-to-positivity values of the corresponding blood cultures (S2 Table).

**Fig 6:**
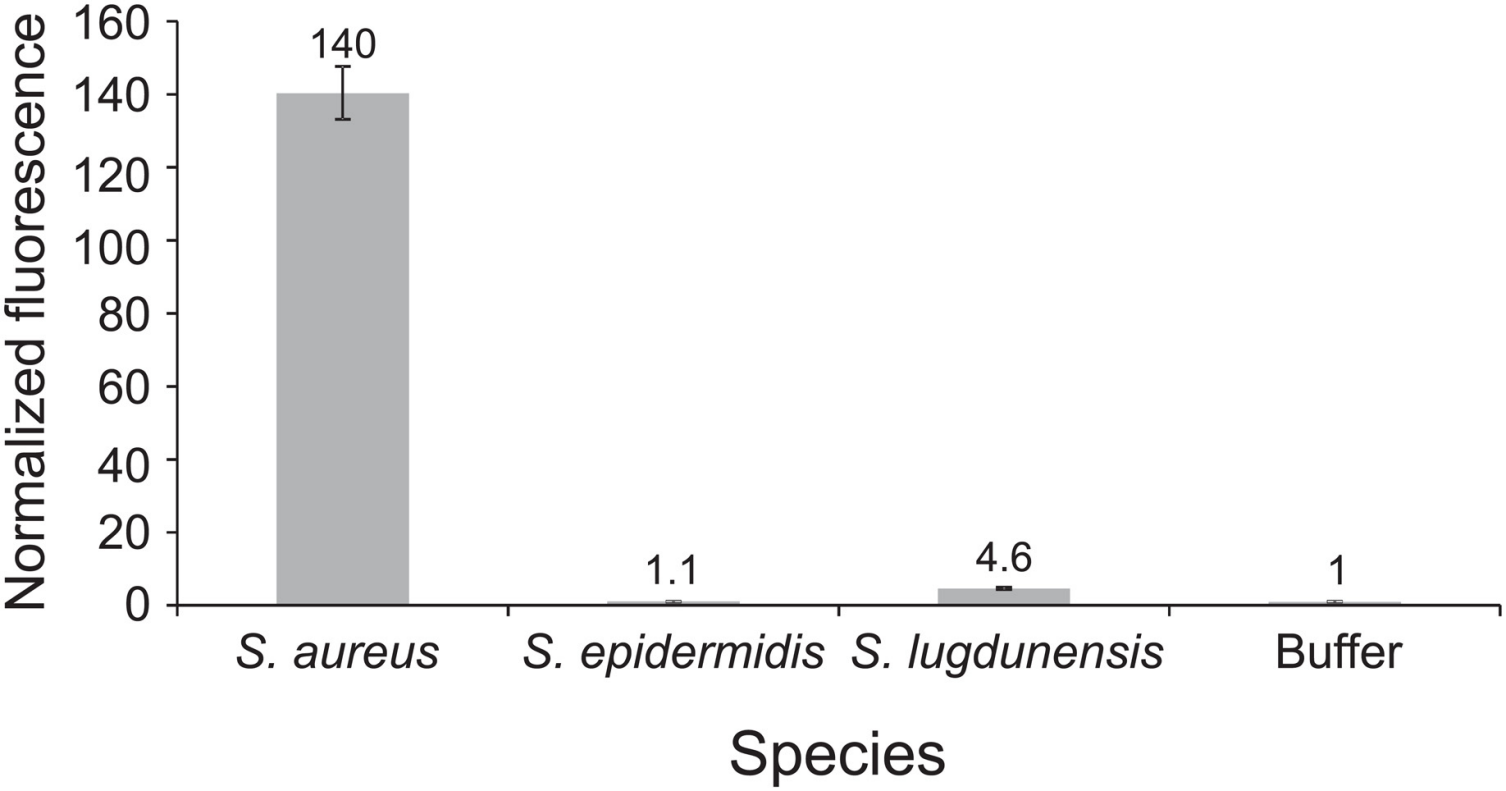
Nuclease activity in culture supernatants of *S*. *aureus*, *S*. *epidermidis* and *S*. *lugdunensis*. An overnight culture supernatant of each species was incubated with the 4mer PolyT ATTO probe for 1 hour at 37°C and fluorescence was measured. Fluorescence values are normalized to that of a control sample in which buffer was substituted for culture supernatant; note normalization values above each bar.

Finally, we recognized that no coagulase-negative staphylococci were found among the non-*S*. *aureus* species in the patient samples. This was primarily due to practical aspects of acquiring salvage samples that matched the criteria we used (see *Clinical Plasma Samples* in Materials and Methods). The fact that repeat blood cultures are always prepared from patients with confirmed SAB, but usually not from patients whose initial cultures yield coagulase-negative staphylococci, made acquisition of *S*. *aureus*-positive blood samples much easier. To gain some insight into the likelihood that coagulase-negative staphylococci might produce positive nuclease assay signals, we measured relative nuclease activities of culture supernatants of *S*. *lugdunensis* (a virulent species that causes disease similar to that of *S*. *aureus* but is typically methicillin susceptible), *S*. *epidermidis* (a common contaminant and cause of hardware-associated infections) and *S*. *aureus* (Fig 6). For this, we used the same probe as for the assay shown in Fig 5. The low nuclease activity levels we found in *S*. *epidermidis* and *S*. *lugdunensis* supernatants suggest that these species will not likely yield positive nuclease assay signals.

## Discussion

In this proof-of-concept study, we describe the development and initial validation of a rapid and inexpensive means of detecting SAB, a common condition with a high mortality rate [3]. We tested our approach on a total of 17 human plasma samples from individuals with culture assay-confirmed SAB; 16 of these yielded greater signals in our rapid nuclease assays than all of the corresponding control samples, suggesting a high degree of assay sensitivity. The lower signals observed for the 47 total control samples, which included 16 confirmed non-bacteremic, 9 presumed non-bacteremic and 22 confirmed bacteremic with a non-*S*. *aureus* species (16 with same day confirmation plus 6 confirmed bacteremic 1 day prior), indicate a high degree of assay specificity. Our 3 hour assay for detecting MN directly from patient plasma entailed three basic steps: 1) heat treatment that inactivates MN-inhibitory antibodies present in plasma; 2) concentration of MN via immunoprecipitation with an antibody that does not inhibit MN activity while bound; and 3) detection of MN enzymatic activity with a hypersensitive, optimized, activatable fluorescent probe. The resulting detection sensitivity for MN in plasma (i.e., within the attomolar range) is unprecedented, to the best of our knowledge.

The background-level nuclease assay signals obtained for the plasma samples from individuals with confirmed non-*S*. *aureus* bacteremia indicates that the nuclease assay is unaffected by general immune responses to bacteremia. Other potential sources of false positive signals include nucleases from non-*S*. *aureus* bacteria. Many of these species are rarely found in blood cultures; a comprehensive evaluation of assay specificity is therefore impossible with the limited sample numbers included in this proof-of-concept study. In the event that non-*S*. *aureus* pathogens (e.g., other staphylococci) are subsequently found to yield false-positive nuclease assay signals, a monoclonal antibody that selectively inhibits MN can likely be incorporated in this assay to differentiate between *S*. *aureus* and alternative pathogens [23].

The lack of rapid methods for diagnosing bacteremia and identifying the responsible pathogen is likely rooted in the exceedingly low concentration of bacteria in the blood of bacteremic individuals [8]. Historically, the difficulty of measuring such low numbers of bacteria has been addressed by amplifying the number of bacteria present in patient samples via culture. A disadvantage of this approach is that it is time-consuming. Current microbiological laboratory methods for diagnosing SAB depend on an initial blood culturing step that takes a day or longer (e.g., see time-to-positivity values in S1 and S2 Tables). *S*. *aureus* is typically identified from agar-plated bacterial colonies (these are grown as 18–24 hour subcultures from positive blood culture bottles) with biochemical methods (e.g., measurement of coagulase activity [17]) that have been used for decades. Methods developed more recently for *S*. *aureus* identification include PCR- and mass spectrometry- based approaches that eliminate subculture on plates, but not broth-based blood culture. Although these methods can save time, the total time remains lengthy due to their dependence on the initial culturing step. These technologies are also expensive and typically require sophisticated instrumentation, thus limiting their availability to larger clinical diagnostic laboratories.

In contrast, the MN assay we developed requires a minimal laboratory infrastructure, with the most sophisticated instrument being a fluorescence plate-reader. While the assay could be carried out in many smaller clinical diagnostic laboratories, it can also likely be automated with existing equipment found in larger clinical chemistry laboratories. We anticipate the cost of materials to process a plasma sample to be less than $10, with savings beyond this likely with scale-up production of the reagents. This number compares favorably with the high cost of treating patients with SAB [24]; substantial cost savings can be expected for these patients if their condition is diagnosed rapidly and appropriate therapy is begun much earlier.

We do not envision that assays for MN activity will replace existing methods for bacteremia diagnosis because MN assays are pathogen-specific (i.e., they will not detect other species). However, the near-unique status of *S*. *aureus* as a common and especially problematic pathogen (e.g., high virulence and metastatic propensity) provides a strong rationale for sufficiently sensitive *S*. *aureus* specific assays when bacteremia is suspected. We note that our assay for MN is not expected to distinguish between bacteremia with methicillin-sensitive *S*. *aureus* (MSSA) versus that with methicillin-resistant *S*. *aureus* (MRSA). However, in regions where MRSA is prevalent, a positive nuclease assay result would be a strong indication that a patient may have MRSA and would thus lead to early administration of antibiotics known to effectively treat it and termination of antibiotic coverage for other potential pathogens. In the event that subsequent assays determine the *S*. *aureus* strain is MSSA, this treatment could be changed to an optimally effective beta lactam antibiotic. In addition to its use as a tool for initial SAB diagnosis, our MN detection assay could be used to follow the efficacy of SAB treatment; an assay turnaround time of 3 hours (versus a day or longer) could enable much more rapid adjustments to ineffective antibiotic therapies.

We are uncertain of the reason for the lack of a clear correspondence between the signal observed in the nuclease assays and the time-to-positivity values of the corresponding culture-based clinical assays. One possibility is that nuclease levels in plasma may be determined by factors in addition to the number of viable bacteria in the blood. For instance, a patient may be bacteremic as a result of a focal infection, such as endocarditis; secretion of MN from the bacteria present in the focal infection might then lead to a greater MN blood level than would result solely from the bacteria present in the blood circulation. This possibility will require further study to verify, but it seems conceivable that the signal strength for the MN assays may provide valuable diagnostic data in addition to a simple indication of bacteremia.

In summary, we have developed a rapid, inexpensive and ultrasensitive assay for MN that detects the presence of this *S*. *aureus* enzyme in the blood of individuals with confirmed SAB. Our results suggest this assay will be highly specific and sensitive for SAB. An essential next step needed to evaluate this assay prior to clinical applicability is a clinical study including sufficient numbers of patients to precisely define the assay specificity and sensitivity. Considering that the assay provides a potential means to address an important unmet need for a common and costly clinical problem that causes substantial morbidity and mortality, such a follow up study seems warranted.

## Materials and Methods

### Study Design

To evaluate the MN detection assay with clinical plasma samples drawn from bacteremic and non-bacteremic individuals, plasma samples drawn for clinical diagnostic assays, but no longer needed for patient care, were selected, salvaged and de-identified by the Tissue Procurement Core (TPC) facility prior to transfer to our research team for processing. The MN detection assay was evaluated with an initial protocol in which 660 μl of each plasma sample was used as input, and a second protocol in which 2 ml of each plasma sample was used as input. The plasma samples for the first protocol were frozen when they were received and thawed immediately prior to being used in the assay; plasma samples for the second protocol were processed without freezing. Plasma samples were evaluated for nuclease activity in batches. A control sample which included buffer in place of serum was included with each batch. The fluorescence levels of the plasma samples were normalized to these control fluorescence levels (see figure legends) and combined to yield the compilations shown. Some batches processed with the second protocol are not included in the data compilation due to failed control reactions. Culture assay confirmed *S*. *aureus* positive plasma samples for which same day culture assay data are unavailable (i.e., culture assay and nuclease assay blood draws were not on the same day) are also not included due to the difficulty in interpreting these data. The researcher carrying out the nuclease assays was blinded to the results of the culture-based assays when carrying out the second protocol (see *Clinical Plasma Samples* for details).

### Quenched Fluorescent Oligonucleotide Probes

Oligonucleotide probes were synthesized and HPLC purified by Integrated DNA Technologies (IDT) of Coralville, IA. Upon receipt of lyophilized probes from IDT, the probes were either stored directly at -80°C or dissolved in TE (10 mM Tris-HCl pH 8.0 and 1 mM EDTA, Ambion) for a final concentration of 500 μM, aliquoted into 1 μl volumes, and then stored at -80°C.

### Heat Treatment of Human Plasma and Serum Spiked with Purified MN

We used pooled human serum or plasma (lithium heparin anticoagulant, no filtration) from Bioreclamation IVT and purified MN from Worthington to evaluate the thermostability of MN, non-target nucleases and inhibitory antibodies in human blood. To enable MN thermostability, CaCl_2_ (from a 1 M stock solution, Sigma) was added to the plasma, yielding a final concentration of approximately 10 mM. Because the plasma used here was not infected, it did not initially contain any MN. Prior to addition to plasma, the pure nuclease was pre-diluted into 50 mM Tris-HCl pH 9.0, 10 mM CaCl_2_ from a 10 unit/μl stock solution (stock buffer consisted of 50% glycerol, 50% DPBS without divalent cations(Gibco)) in 1.5 ml low protein binding microfuge tubes (Eppendorf). These MN dilutions in buffer were diluted 1:100 in the plasma and calcium mixture to yield the experimental concentrations. A “no nuclease” control sample was prepared with a 1:100 dilution of 50 mM Tris-HCl pH 9.0, 10 mM CaCl_2_ buffer in the plasma and calcium mixture. Next, with the exception of experiments that assess the inactivation of inhibitory antibodies, samples were placed in a 90°C heat block for 20 minutes. For assessment of inactivation of inhibitory antibodies, samples of diluted MN in serum or plasma and controls were divided in half, with one half subjected to the heating protocol and the other half reserved as unheated controls. The unheated control samples were stored at 4°C during the heating protocol, and the other samples were incubated in a 90°C heat block for 20 minutes. For determination of the time needed to inactivate the inhibitory antibodies, samples with equivalent nuclease concentrations were incubated in 90 or 100°C heat blocks for 2, 5, 10, 12, 15, and 20 minutes with an additional sample kept at room temperature. Then, for all experiments, heated plasma and serum samples, which became cloudy upon heating due to protein precipitation, were then centrifuged at 17,000×g for 10 minutes. Supernatants were transferred to fresh tubes and used for the subsequent immunoprecipitation and/or nuclease activity assay.

### Heat Treatment of Plasma Samples from *S*. *aureus* Bacteremic Patients and Control Subjects

Heparinized plasma specimens obtained from patients with culture-confirmed SAB, culture-confirmed bacteremia of another species, culture-confirmed negative for bacteremia, or from individuals exhibiting no signs of active infections (no culture performed), were provided by the University of Iowa Tissue Procurement Core Facility. These specimens were tested immediately or aliquoted and stored at -80°C. For the assays that used frozen specimens (i.e., the initial clinical specimen experiment), aliquots of each specimen were thawed at 25°C and combined into a 660 μl sample in a 1.5 ml low protein binding microfuge tube. For the assays that used fresh specimens (i.e., the second clinical specimen experiment), 2 ml of plasma was divided equally into two 1.5 ml low protein binding microfuge tubes. To enable MN thermostability, CaCl_2_ (from a 1 M stock solution, Sigma) was added to the plasma, yielding a final concentration of approximately 10 mM CaCl_2_. Samples were then placed in a 90°C heat block for 20 minutes. The plasma samples, which became cloudy upon heating due to protein precipitation, were then centrifuged at 17,000×g for 10 minutes. Supernatants were transferred to fresh tubes and used for the subsequent immunoprecipitation/nuclease assay.

### Immunoprecipitation of MN from Plasma

Protein G-coupled magnetic beads (Life Technologies) were resuspended in the manufacturer’s vial by rotating the vial at room temperature for 5 minutes. For each plasma sample, 40 μl of the beads suspension was added to an empty 1.5 ml low protein binding microfuge tube. An additional tube was prepared in the same way in parallel for use as a “no plasma” control. The tubes were placed on a magnet for ~1 minute to separate the beads from the manufacturer’s storage solution, and the solution was removed with a pipette. The beads were resuspended in 1 ml of 0.04% Tween-20 (Amresco) in DPBS without divalent cations (wash buffer). The tubes were then placed on the magnet to separate the beads from the wash buffer, and the buffer was removed with a pipette. This washing step (re-suspending beads in wash buffer and removing wash buffer) was repeated once for a total of two washes. Approximately 37 μg of anti-MN rat monoclonal antibody (isotype IgG2a, Pierce Biotechnology, Inc. custom produced antibody, stored in 50% glycerol at -20°C or in 20 mM K_3_PO_4_, 150 mM NaCl, 0.02% NaN_3_, pH 7.2–7.4 buffer at 4°C) was diluted to 200 μl with wash buffer and used to resuspend the beads in each beads-containing tube. The tubes were incubated with beads and antibody on a rotator at room temperature for 15 min, centrifuged briefly at very low speed (less than 1 second at less than 100×g), and then placed on the magnet to separate the beads from the antibody solution. The solution was removed with a pipette. The beads were then washed with wash buffer twice as described above. Next, each heat-processed human plasma supernatant (prepared as described in *Heat Treatment of Human Plasma and Serum Spiked with Purified MN* or *Preparation of Plasma Samples from S*. *aureus Bacteremic Patients and Control Subjects*) was added to an antibody/beads-containing tube. For *S*. *aureus* bacteremic and presumed-negative control specimens, the entire supernatant from each of these samples was used; the volume of each supernatant was approximately half of the sample’s volume before heat precipitation and varied by ±5% between samples. This volume was limited to no more than half of the sample’s pre-heat precipitation volume for the second patient specimen experiment. A volume equal to half of each sample’s initial volume (1 ml for 2 ml samples or 330 μl for 660 μl samples) of wash buffer was added to the beads-containing tube reserved for the “no plasma” control. For the side-by-side comparison of the assay sensitivity with and without immunoprecipitation, 500 μl or 1 ml of each supernatant was used, each being half of the volume of the sample before heat precipitation. Following the resuspension of antibody-coupled beads with heat-processed supernatants or buffer solution, the tubes were incubated on a rotator at room temperature for 1 hour. Next, the tubes were centrifuged briefly at very low speed (less than 1 second at less than 100×g) and then placed on a magnet to separate the beads from the heat-processed plasma supernatants or buffer solution, and the solutions were removed with a pipette. The beads were then washed with 1 ml of wash buffer for samples with an initial plasma volume of less than 2 ml or with 1 ml of 0.04% Tween-20 in DPBS with divalent cations (Gibco) for assays with an initial plasma volume of 2 ml as described above a total of 3 times, using a fresh low protein binding tube for each wash. The beads were then washed twice with 1 ml of 50 mM Tris-HCl pH 9.0, 10 mM CaCl_2_.

### Fluorescence Plate-reader Assay for Samples without Immunoprecipitation

For experiments in which no immunoprecipitation was carried out, probe incubation reactions and plate reader measurements were carried out as follows. 1 μl of probe (11mer PolyT FAM labeled probe unless otherwise indicated) diluted to a concentration of 50 μM in 50 mM Tris-HCl pH 9.0, 10 mM CaCl_2_, was added to 9 μl of each sample (heat-processed human serum supernatant, unheated control human serum sample, or buffer, each containing samples with and without nuclease). The tubes were incubated in the dark at 37°C for 1 hour. To stop the digestion by MN, which requires calcium for activity, 290 μl of 10 mM EDTA (prepared from 0.5 M stock, Ambion) and 10 mM EGTA (prepared from 0.5 M stock, Bio-World) in DPBS without divalent cations, was added to each tube. The stopped reactions were mixed by pipetting, and then 90 μl was transferred to each of 3 wells of a 96-well black polystyrene plate (Thermo Scientific) for triplicate readings. Fluorescence was measured in a fluorescence plate-reader (Analyst HT or Biotek Synergy Mx) at 485/530 nm excitation/emission.

### Fluorescence Plate-reader Assay for Samples with Immunoprecipitation

Probe incubation reactions and plate-reader measurements of immunoprecipitated nuclease samples were carried out as follows. Each bead sample (prepared as described in *Immunoprecipitation of MN from Plasma*) was resuspended in 60 μl 50 mM Tris-HCl pH 9.0, 10 mM CaCl_2_. 1 μl of probe (4mer PolyT ATTO-488 labeled probe or 11mer PolyT FAM labeled probe, as indicated) diluted to a concentration of 50 μM in 50 mM Tris-HCl pH 9.0, 10 mM CaCl_2_, was then added to each beads suspension. The tubes were incubated on a rotator at room temperature in the dark for 1 hour. The tubes were centrifuged briefly at very low speed (less than 1 second at less than 100×g) to spin the contents to the bottom of the tubes, placed on a magnet, separating beads from probe solution, and then 50 μl of each probe-containing supernatant was transferred to a well of a 96-well black polystyrene plate (Thermo Scientific). Fluorescence was measured in a fluorescence plate-reader (Analyst HT or Biotek Synergy Mx) at 485/530 nm excitation/emission.

### MN Stability

To compare the activity of MN in plasma stored at 4°C for three days with freshly prepared MN/plasma dilutions, we used human plasma pooled from healthy donors combined with defined amounts of pure MN. Prior to addition to plasma, pure MN was pre-diluted into 50 mM Tris-HCl pH 9.0, 10 mM CaCl_2_ from a 10 unit/μl stock solution in 1.5 ml low protein binding microfuge tubes. These MN dilutions in buffer were diluted 1:100 in plasma to yield the experimental concentrations. A “no nuclease” control sample was prepared with a 1:100 dilution of 50 mM Tris-HCl pH 9.0, 10 mM CaCl_2_ buffer in plasma. Samples were then stored at 4°C for three days. On the third day, a fresh set of nuclease dilutions was prepared in plasma exactly as described above. To enable MN thermostability, CaCl_2_ (from a 1 M stock solution, Sigma) was added to the plasma samples, yielding a final concentration of approximately 10 mM. These fresh and third day samples were tested for nuclease activity as described in *Heat Treatment of Human Plasma and Serum Spiked with Purified MN* and *Fluorescence Plate-reader Assay for Samples without Immunoprecipitation*.

### Probe Optimization

For selection of the optimally sensitive probe, nuclease assays were carried out with defined amounts of pure MN diluted in 50 mM Tris-HCl pH 9.0, 10 mM CaCl_2_. The following probes were tested as described in *Fluorescence Plate-reader Assay for Samples without Immunoprecipitation*: 11mer and 4mer PolyT FAM labeled probes and 11mer and 4mer PolyT ATTO-488 labeled probes.

### Blood culture

Standard-of-care blood culture was performed at the University of Iowa Hospitals and Clinics Clinical Microbiology Laboratory. Blood culture bottles (BD Bactec Plus Aerobic/F and Lytic 10/Anaerobic F) were incubated on a Bactec FX unit and time to positivity for each bottle was reported from within the associated BD Epicenter software (Becton Dickinson; Franklin Lakes, New Jersey USA). Bacterial identifications were made with the Bruker BioTyper system [version 4.0.0.1 (5627); Bruker Daltonics, Billerica, MA USA] from subcultures of positive bottles to standard agar media.

### Clinical Plasma Samples

Excess human plasma samples, drawn for clinical diagnostic assays but no longer required for patient care, were identified by personnel of the University of Iowa Tissue Procurement Core (TPC) facility and released by the University of Iowa Clinical Chemistry Laboratory to be provided to our research team following de-identification by the TPC. Once a patient with positive blood culture information was identified by the Clinical Microbiology Laboratory and the TPC was notified, salvage chemistry plasma samples from the identified patient that had been drawn on the same day (or one day prior, in some cases) were selected by TPC personnel and requested for release from the chemistry lab. Sample selection criteria also included the result of the blood culture because inclusion of samples from each of the categories previously described was essential for evaluating the nuclease assay. TPC personnel provided samples to our research team blinded with respect to the associated culture results. Where indicated (see figure legends), the samples were unblinded by TPC personnel only after the nuclease assays were completed (by E.B.) and results reviewed (by E.B. and J.M.). Criteria for selection of “presumed uninfected” plasma controls included normal creatinine levels, no indications of infection and blood draw dates (control and test samples were equivalently old).

### Assessment of Nuclease Activity in Culture Supernatants

Overnight cultures of *S*. *aureus* (strain: ATCC 25923), *S*. *epidermidis* (strain: AH2490) and *S*. *lugdunensis* (strain: AH2160) were grown overnight in tryptic soy broth at 37°C with shaking. *S*. *epidermidis* and S. *lugdunensis* strains were kindly provided by Dr. Alex Horswill of the University of Iowa. Dilutions of each supernatant were prepared in 50 mM Tris-HCl, pH 8.0, 10 mM CaCl_2_; 9 μl of each was then combined with 1 μl of 50 μM 4mer PolyT (ATTO-488 labeled) for each reaction. Final supernatant dilutions were 1:200 in each reaction. Reactions were prepared and fluorescence was measured as detailed in *Fluorescence Plate-reader Assay for Samples without Immunoprecipitation*.

### Ethics statement

The study format for using human plasma samples (see Materials and Methods for description) was reviewed by the chair of the University of Iowa Institutional Review Board (#201403717 and #201503727) who determined that it did not meet the regulatory definition of human subjects research. Patient consent was not obtained because the work was limited to laboratory assessment of de-identified clinical samples that were collected for clinical diagnostic assays, but no longer needed for patient care. The samples were provided by the University of Iowa Clinical Chemistry Laboratory (http://www.medicine.uiowa.edu/pathology/patientcare/Clinical_Core_Laboratories/).

## Supporting Information

S1 Table Blood culture results for individuals whose plasma was used in nuclease assay of Fig 3B. Blood was drawn for blood cultures on the same day as the blood that was used for nuclease assays. “Time-to-positivity” indicates the time elapsed for the blood cultures to indicate bacterial growth. Note: both aerobic and anaerobic cultures were prepared. In cases where only one of these became positive, only the positive value is included. “Presumed negative” indicates that these specimens were drawn from individuals who were not exhibiting signs of active infections; blood cultures were not prepared from these individuals. Plasma samples were stored at 4°C for the number of days indicated in the “Age of Sample” column prior to being frozen for storage. Frozen samples were processed with the nuclease activity assay immediately after thawing.
(PDF)

S2 Table Blood culture results pertaining to plasma samples used in nuclease assay of Fig 5. “Time-to-positivity” indicates the time elapsed for the blood cultures to indicate bacterial growth. Note: both aerobic and anaerobic cultures were prepared. In cases where only one of these became positive, only the positive value is included. Plasma samples were stored at 4°C for the number of days indicated in the “Age of Sample” column prior to being used (without freezing) in the nuclease activity assay. Blood was drawn for blood cultures on the same day, or one day prior to the blood that was used for nuclease assays, as indicated in the far right column. Samples are listed (from top to bottom) in the same order as they appear in the figure (from left to right).
(PDF)
