# Supplementary material for: Rapid, Culture-Free Detection of Staphylococcus aureus Bacteremia
Source: PLoS One. 2016 Jun 15;11(6):e0157234. doi: 10.1371/journal.pone.0157234 (PMC4909304; doi:10.1371/journal.pone.0157234)
Supplement: S1 Table — Blood was drawn for blood cultures on the same day as the blood that was used for nuclease assays. “Time-to-positivity” indicates the time elapsed for the blood cultures to indicate bacterial growth. Note: both aerobic and anaerobic cultures were prepared. In cases where only one of these became positive, only the positive value is included. “Presumed negative” indicates that these specimens were drawn from individuals who were not exhibiting signs of active infections; blood cultures were not prepared from these individuals. Plasma samples were stored at 4°C for the number of days indicated in the “Age of Sample” column prior to being frozen for storage. Frozen samples were processed with the nuclease activity assay immediately after thawing. (PDF) [file pone.0157234.s001.pdf]

S1 Table

Sample ID	Time-to-positivity (Aerobic)	Time-to-positivity (Anaerobic)	Age of Sample (Days stored at 4°C)
K	15 hr, 37 min	15 hr, 37 min	6
G	17 hr, 53 min		4
J		19 hr, 15 min	4
M	20 hr, 11 min		3
P	20 hr, 15 min		4
V	29 hr, 28 min		5
B	29 hr, 53 min		5
D	33 hr, 26 min		5
U	58 hr, 25 min		7
A	Presumed negative	Presumed negative	5
F	Presumed negative	Presumed negative	3
H	Presumed negative	Presumed negative	4
I	Presumed negative	Presumed negative	4
L	Presumed negative	Presumed negative	6
N	Presumed negative	Presumed negative	3
R	Presumed negative	Presumed negative	7
S	Presumed negative	Presumed negative	5
T	Presumed negative	Presumed negative	5

S1 Table. Blood culture results for individuals whose plasma was used in nuclease assay of **Figure 3B**. Blood was drawn for blood cultures on the same day as the blood that was used for nuclease assays. "Time-to-positivity" indicates the time elapsed for the blood cultures to indicate bacterial growth. Note: both aerobic and anaerobic cultures were prepared. In cases where only one of these became positive, only the positive value is included. "Presumed negative" indicates that these specimens were drawn from individuals who were not exhibiting signs of active infections; blood cultures were not prepared from these individuals. Plasma samples were stored at 4°C for the number of days indicated in the "Age of Sample" column prior to being frozen for storage. Frozen samples were processed with the nuclease activity assay immediately after thawing.
