# Supplementary material for: Rapid, Culture-Free Detection of Staphylococcus aureus Bacteremia
Source: PLoS One. 2016 Jun 15;11(6):e0157234. doi: 10.1371/journal.pone.0157234 (PMC4909304; doi:10.1371/journal.pone.0157234)
Supplement: S2 Table — “Time-to-positivity” indicates the time elapsed for the blood cultures to indicate bacterial growth. Note: both aerobic and anaerobic cultures were prepared. In cases where only one of these became positive, only the positive value is included. Plasma samples were stored at 4°C for the number of days indicated in the “Age of Sample” column prior to being used (without freezing) in the nuclease activity assay. Blood was drawn for blood cultures on the same day, or one day prior to the blood that was used for nuclease assays, as indicated in the far right column. Samples are listed (from top to bottom) in the same order as they appear in the figure (from left to right). (PDF) [file pone.0157234.s002.pdf]

S2 Table

Sample ID	Pathogen	Time-to-positivity (Aerobic)	Time-to-positivity (Anaerobic)	Age of Sample (Days stored at 4°C)	Days Elapsed between blood culture and nuclease assay blood draws
042115_1	*Enterobacter cloacae* complex, *Streptococcus anginosus*	43 hr, 50 min	3 hr, 56 min	3	1
041515_1	*Escherichia coli*	13 hr, 24 min		2	1
042915_4	*Escherichia coli*	58 hr, 18 min	58 hr, 18 min	3	1
042215_4	Positive Gram Stain, *Escherichia coli*		35 hr, 13 min	1	1
040915_9	*Klebsiella pneumoniae*	40 hr, 47 min		4	1
042315_2	*Streptococcus pneumoniae*	24 hr, 55 min		2	1
042915_1	*Burkholderia cepacia* complex	41 hr, 35 min		5	0
040915_8	*Enterococcus faecalis*	54 hr, 50 min	34 hr, 49 min	3	0
040915_11	*Enterococcus faecalis*	24 hr, 58 min	25 hr	3	0
050515_2	Positive Gram Stain, *Enterococcus faecalis*	32 hr, 2 min		4	0
042215_3	*Enterococcus faecium*	31 hr, 33 min		4	0
050115_4	*Enterococcus faecium*	50 hr, 31 min	40 hr, 31 min	2	0
042915_3	Vancomycin Resistant *Enterococcus faecium*		51 hr, 45 min	3	0
050115_1	*Escherichia coli*	15 hr, 48 min		4	0
050115_2	*Escherichia coli*	6 hr, 13 min		4	0
050115_5	*Escherichia coli*	10 hr, 23 min		2	0
050515_5	*Escherichia coli*		16 hr, 16 min	3	0
050715_1	*Klebsiella pneumoniae*	24 hr, 35 min		3	0
050715_3	*Proteus mirabilis*	14 hr, 10 min	14 hr, 11 min	2	0
041615_2	*Pseudomonas aeruginosa*	46 hr, 31 min		3	0
042115_2	*Pseudomonas aeruginosa*	6 hr, 15 min		3	0
040915_10	*Pseudomonas aeruginosa*, mucoid	47 hr		5	0
040915_4	*S. aureus*	25 hr, 16 min		3	0
042215_1	*S. aureus*	20 hr, 46 min		4	0
042915_2	*S. aureus*		41 hr, 48 min	3	0
042915_7	*S. aureus*		3 hr, 56 min	2	0
050115_3	*S. aureus*	41 hr, 44 min	31 hr, 44 min	3	0
050515_1	*S. aureus*	15 hr, 27 min	45 hr, 27 min	5	0
050515_3	*S. aureus*	34 hr, 12 min	16 hr, 13 min	3	0
050715_4	*S. aureus*	4 hr, 53 min		2	0

S2 Table. Blood culture results pertaining to plasma samples used in nuclease assay of **Figure 5**. “Time-to-positivity” indicates the time elapsed for the blood cultures to indicate bacterial growth. Note: both aerobic and anaerobic cultures were prepared. In cases where only one of these became positive, only the positive value is included. Plasma samples were stored at 4ºC for the number of days indicated in the “Age of Sample” column prior to being used (without freezing) in the nuclease activity assay. Blood was drawn for blood cultures on the same day, or one day prior to the blood that was used for nuclease assays, as indicated in the far right column. Samples are listed (from top to bottom) in the same order as they appear in the figure (from left to right).
